# Study on the fermentation effect of *Rhodotorula glutinis* utilizing tofu whey wastewater and the influence of *Rhodotorula glutinis* on laying hens

**DOI:** 10.3389/fnut.2023.1125720

**Published:** 2023-02-24

**Authors:** Xifei Xu, Wenjian Liu, Honghong Niu, Mei Hua, Ying Su, Xinyu Miao, Yanping Chi, Hongyan Xu, Jinghui Wang, Mubai Sun, Da Li

**Affiliations:** ^1^Department of Food Science and Engineering, College of Agriculture, Yanbian University, Yanji, China; ^2^Laboratory of Food Microbiology, Institute of Agro-product Process, Jilin Academy of Agricultural Science, Changchun, China; ^3^Department of Microbiology, College of Life Sciences, Jilin Normal University, Siping, China

**Keywords:** tofu whey wastewater, *Rhodotorula glutinis*, carotenoids, optimization of fermentation conditions, nutrient composition analysis

## Abstract

**Background:**

Tofu whey wastewater (TWW) is the wastewater of tofu processing, which is rich in a variety of nutrients. *Rhodotorula glutinis* can make full use of TWW to ferment and reproduce yeast cells, produce carotenoids and other nutrients, improve the utilization value of TWW, and reduce environmental pollution and resource waste.

**Methods:**

In this study, the nutrient composition changes of TWW treated by *Rhodotorula glutinis* were analyzed to reformulate TWW medium, and the optimal composition and proportion of TWW medium that can improve the biomass and carotenoids production of *Rhodotorula glutinis* were explored. Meanwhile, the *Rhodotorula glutinis* liquid obtained under these conditions was used to prepare biological feed for laying hens, and the effect of *Rhodotorula glutinis* growing on TWW as substrate on laying performance and egg quality of laying hens were verified.

**Results:**

The results showed that the zinc content of TWW after *Rhodotorula glutinis* fermentation increased by 62.30%, the phosphorus content decreased by 42.31%, and the contents of vitamin B1, B2 and B6 increased to varying degrees. The optimal fermentation conditions of *Rhodotorula glutinis* in the TWW medium were as follow: the initial pH was 6.40, the amount of soybean oil, glucose and zinc ions was 0.80 ml/L, 16.32 g/L, and 20.52 mg/L, respectively. Under this condition, the biomass of *Rhodotorula glutinis* reached 2.23 g/L, the carotenoids production was 832.86 μg/g, and the number of effective viable yeast count was 7.08 × 10^7^ cfu/ml. In addition, the laying performance and egg quality of laying hens fed *Rhodotorula glutinis* biological feed were improved.

**Discussion:**

In this study, we analyzed the composition changes of TWW, optimized the fermentation conditions of *Rhodotorula glutinis* in TWW medium, explored the influence of *Rhodotorula glutinis* utilizing TWW on laying layers, and provided a new idea for the efficient utilization of TWW.

## 1. Introduction

TWW is a by-product in the production process of soybean products, rich in soybean whey protein, polypeptide, oligosaccharides, isoflavones, and other nutrients (1, 2). At present, TWW is usually discharged as waste, with loss of protein resources and causing environmental pollution (3, 4). The treatment of TWW before discharge is complicated and expensive (5). Therefore, the efficient utilization of TWW by physical, chemical methods and biological methods has attracted the attention of experts and scholars (5, 6). Soy whey is rich in nutrients and can be used as a growth substrate for some microorganisms (7). Nursyirwani et al. found that *Bacillus toyonensis* increased exponentially in a substrate containing 12% TWW within 24 h (8). Rusydi et al. found that Cyanobacteria *Nostocmuscorum* grew well in TWW medium at a concentration of 40% (9). Ajijah et al. showed that *Chlorella pyrenoidosa* had the highest carotenoids and chlorophyll production at a TWW concentration of 5% and the highest medium density and growth rate at a TWW concentration of 20%, while *Arthrospira platensis* had the highest carotenoids content at a TWW concentration of 10% (10). A variety of metabolites beneficial to humans can be obtained by microbial fermentation of TWW (7). *Weissella hellenica D1501* fermentation of soy whey can protect nerve cells from potential oxidative damage (11). *Lactobacillus acidophilus* fermented soy whey produces antihypertensive active substances (12). Soybean whey shows a prebiotic effect and improves the mineral balance, especially calcium and magnesium (1).

*Rhodotorula glutinis* is a good source of protein, lipids, and vitamins in animal feed (13). *Rhodotorula glutinis* produces a variety of beneficial substances, such as carotenoids, which can be used as antioxidants to enhance animal immunity, so it is commonly used as biological feed additives (14, 15). Carotenoids play a role in deepening the color of egg yolk, and can also improve their oxidative stability in the breeding of laying hens (16, 17). The addition of *Rhodotorula glutinis* to the diet improved hen laying performance and egg quality by Sun et al. (18). Hu et al. found that oral administration of *Rhodotorula glutinis* improved the growth performance of piglets, enhanced their antioxidant capacity and gastrointestinal digestion capacity, and maintained the intestinal microbial balance of piglets (19). *Rhodotorula glutinis* was able to metabolize different substances as sources of carbon and nitrogen, such as the use of olive oil waste as a cheap substrate to produce carotenoids, and the use of carbon sources in sugar beet pulp to produce lipids and carotenoids (20, 21). *Rhodotorula glutinis*, can also use nutrients in TWW, its yield is closely related to the medium substrate species. Therefore, the optimization of TWW medium components is expected to significantly improve the biomass and carotenoids production of *Rhodotorula glutinis*.

There have been many reports on the use of TWW to produce active substances, but there are few reports on the use of TWW as a fermentation substrate to improve the biomass and carotenoids yield and to explore the composition of TWW before and after fermentation and its effect on laying hens (4, 22, 23). In this study, the full utilization of the TWW and the large increase of the biomass and carotenoids production of the *Rhodotorula glutinis* were realized. Furthermore, in this work, the composition of untreated TWW and *Rhodotorula glutinis* were analyzed, and the effect of *Rhodotorula glutinis* using TWW on laying performance and egg quality of laying hens were studied.

## 2. Materials and methods

### 2.1. Materials and reagents

The *Rhodotorula glutinis SRY* strain was isolated and maintained by the Food Microbiology Team, Agro-Processing Institute, Jilin Academy of Agricultural Sciences. Protein, yeast extract powder, agar powder, sodium chloride, glucose, concentrated hydrochloric acid, acetone, zinc sulfate, manganese sulfate, ferrous sulfate, copper sulfate, hydrogen peroxide and sodium bicarbonate were all purchased from Sangon Biotech (Shanghai) Co., Ltd.; vitamin B1 was purchased from Cisen Pharmaceutical Co., Ltd.; soybean oil was purchased from Yihai Kerry Arawana Holdings Co, Ltd. and TWW is taken from the local market.

### 2.2. Preparation of the liquid culture

The LB liquid culture medium was formulated as follows: peptone (10.0 g/L), yeast extract (3.0 g/L), glucose (3.0 g/L), and NaCl (5.0 g/L). The pH of the medium was adjusted to 6.2 and then sterilized at 121°C for 21 min.

The LB solid culture medium was formulated as follow: peptone (10.0 g/L), yeast extract (3.0 g/L), glucose (3.0 g/L), NaCl (5.0 g/L), and agar powder (15.0 g/L). The pH of medium was adjusted to 6.2 and then sterilized at 121°C for 21 min.

The *Rhodotorula glutinis* was melted at room temperature and transferred to LB medium (180 rpm, 30°C for 48 h) to obtain the activated *Rhodotorula glutinis* culture. The activated *Rhodotorula glutinis* culture was then added to LB medium at 10% (v/v), and incubated at 30°C, 180 rpm for 48 h. The viable yeast count of *SRY* (*Rhodotorula glutinis SRY*) was 1.3 × 10^7^ cfu/ml.

### 2.3. Determination and analysis of nutrient composition of TWW

The *Rhodotorula glutinis SRY* seed medium was added to YWW medium at 5% (v/v) and incubated at 180 rpm, 30°C for 48 h. The supernatant was collected by centrifugation (4,000 rpm, 15 min) and the yeast cells were filtered out through a sterile filter (0.22 μm).

The protein content, fat content, reducing sugar content, mineral elements, vitamin B1, vitamin B2, vitamin B6 were determined according to the Chinese National Standard Method GB5009.5-2016, GB5009.6-2016, GB5009.7-2016, GB5009.268-2016, GB5009.84-2016, GB5009.85-2016, GB5009.154-2016, respectively (24–30). The free amino acids content was determined according to the method of Li et al. (31). The specific test methods are described in the Supplementary material. Both untreated and *Rhodotorula glutinis* fermented TWW samples were tested by Qingdao Zhongyi Monitoring Co., Ltd.

### 2.4. Optimization of medium composition and fermentation conditions of TWW in *Rhodotorula glutinis*

#### 2.4.1. Single factor experiment of TWW medium composition optimization

The medium was prepared according to factors and levels as shown in Supplementary Table 1 and *R. glutinis SRY* grown. The effects of initial pH value, glucose content, manganese ion content, copper ion content, magnesium ion content, zinc ion content and ferrous iron ion content on *SRY* biomass, carotenoids content and viable yeast count were investigated.

#### 2.4.2. Design of the Plackett-Burman test

Using the biomass, carotenoids content and viable yeast count of *SRY*, eight factors were selected as the initial pH value (A), soybean oil content (B), glucose content (C), hydrogen peroxide content (D), vitamin B1 content (E), manganese ion content (F), magnesium ion content (G) and zinc ion content (H) for Plackett-Burman test. The range of optimal culture conditions for the 8 factors was taken at high (+1) and low (−1) levels, respectively, and the PB test design factors and levels are shown in Supplementary Table 2.

#### 2.4.3. Desirability function-response surface Box–Behnken test design

According to the results of the Plackett-Burman test, the factors that significantly affect the results were selected for analysis, and biomass (Y1), carotenoids content (Y2), and effective viable number (Y3) of *SRY* were used as the response values to find reasonable optimization conditions (Table 1). The desirability function method was used to comprehensively analyze the three response values and optimize the fermentation conditions.

**Table 1:** Design of box-Benhnken experiment.

Factor	−1	0	+1
A-pH	5.5	6	6.5
B-soybean oil (ml/L)	0.6	0.8	1
C-glucose (g/L)	12.5	15	17.5
H-zinc ion (mg/L)	15	20	25

#### 2.4.4. Determination method of biomass and viable yeast count of *Rhodotorula glutinis*

The cell pellet were obtained by centrifugation (4,000 rpm, 15 min) of *Rhodotorula glutinis SRY* broth (1 L), dried to constant weight at 50°C, weighed and biomass was calculated. The viable yeast count of *Rhodotorula glutinis SRY* in the culture medium was determined by solid plate counting methods. Briefly, *Rhodotorula glutinis SRY* seed culture was diluted at appropriate multiples, evenly spread on LB solid medium. The culture was incubated at 30°C for 72 h and the viable yeast counts of *Rhodotorula glutinis SRY* were counted.

#### 2.4.5. Method of determination of carotenoids content

Carotenoids content in *Rhodotorula glutinis* was determined by the hydrochloride acid thermal breakage method, as follows: the liquid culture (30 ml) was centrifuged (4,000 rpm, 15 min). 3 M HCl (30 ml) was added to the yeast cell pellet, shaken for 45 min, and then heated at 100°C for 5 min. The mixture was cooled and centrifuged (4,000 rpm, 15 min). The sterilized water (30 ml) was added to the yeast cell pellet. And the above process was repeated three times. Acetone (10 ml) was added to the yeast cell pellet, shaken for 1 h and centrifuged (4,000 rpm, 15 min) to obtain carotenoids extracts. The absorbance of the carotenoids extracts was measured at 475 nm. Formula for calculating the carotenoids content:


(1) $$W_{c}=A_{\max}\times D\times V÷\left(0.16\times m\right)$$

where W_c_ is carotenoids content of fermentation broth, μg/g; A_max_ is absorbance at 475 nm; D is dilution of sample; V is organic solvent volume for pigment extraction, ml; m is dry yeast mass, g; 0.16 is molar extinction coefficient of carotenoids.

### 2.5. The effect of *Rhodotorula glutinis* utilizing TWW on laying hens

#### 2.5.1. Design of the experimental animals

The Luhua chicken used in the experiment is a local chicken breed bred by Jilin Academy of Agricultural Sciences. Ninety-six 21-week-old Luhua chickens of similar weight were selected and randomly divided into 6 groups of 16 in each group, and grouped as shown in Table 2.

**Table 2:** Groups of laying hens.

Group	Treatment
NC	Basic feed
0.25% Rh	Contain 0.25% *Rhodotorula* in the basic feed
0.5% Rh	Contain 0.5% *Rhodotorula* in the basic feed
1% Rh	Contain 1% *Rhodotorula* in the basic feed
2% Rh	Contain 2% *Rhodotorula* in the basic feed
5% Rh	Contain 5% *Rhodotorula* in the basic feed

The laying hens in each treatment group were raised in a three-layer chicken cage, with each two birds kept in one cage, and a nipple drinking fountain was used to provide drinking water. The daily feed mass of each layer was 150 g and the feed formula was shown in Table 3. The light time of the chicken cages was 16 h, ventilated for 3 min every 30 min, eggs were collected every day, chicken manure was cleaned and disinfected.

**Table 3:** Nutrient composition and content of basic feed for laying hens.

Feed composition	Content	Nutrient composition	Content
Corn (%)	61.20	Dry matter (%)	87.10
Bean pulp (%)	21.50	Metabolic energy (MJ/kg)	11.52
Soybean oil (%)	1	Crude protein (%)	16.02
Stone powder (%)	8	Calcium (%)	3.8
5% Premix (%)	5	Total phosphorus (%)	0.48
Corn gluten meal (%)	3.3	Lysine (%)	0.81
		Methionine (%)	0.42

#### 2.5.2. Determination of laying performance and egg quality

The laying number and egg weight of laying hens were recorded daily, and the laying rate and average egg weight were calculated. Ten eggs were selected from each group every week. Fifteen grade color fans were used to contrast the color of the egg yolk. Protein height was determined using a protein height meter, and the average value was taken based on the protein height and egg weight, and the Haugh unit value was query on the unit table.


(2) $$\mathrm{Haugh unit}=100\mathrm{lg}\left(H-1.7W^{0.37}+7.57\right)$$

where Haugh unit is a quality standard for the internal quality of eggs, and the parameter is expressed as a score between 0 and 100; H is the protein height, mm; W is egg weight, g.

#### 2.5.3. Measurement of carotenoids in eggs

The well-stirred egg yolk (0.5 g) was added to acetone (10 ml), vortexed for 15 min and then centrifuged at 4000 rpm for 15 min. The absorbance value of the supernatant at 475 nm was determined by UV–visible spectrophotometer. Carotenoids content was calculated according to the following formula:


(3) Carotenoids content(μg/g)=A×V/(0.16×W)

where A is absorbance value at 475 nm; V is the volume of acetone being added, ml; 0.16 is molar extinction coefficient of carotene; W is weight of the egg yolk used in the measurement process, g.

### 2.6. Analysis of data

Test data were analyzed and processed using Design-Expert software (Stat-Ease, Inc.) and SPSS software. Group mean values were compared using a one-way analysis of variance and tested for multiple comparisons by the LSD test. Data are presented as mean ± standard deviation (sd), and *p* < 0.05 represents significance.

## 3. Results

### 3.1. Determination of nutrient composition of TWW

TWW is rich in nutrients, and we detected the changes in protein, fat, reducing sugar, vitamin B1, vitamin B2, vitamin B6, amino acids, and mineral elements (Table 4) in the untreated (before fermentation) and *Rhodotorula glutinis* fermented TWW. The test results showed that the *Rhodotorula glutinis* consumed 41.58% of the protein in the TWW, and produced more vitamin B1, vitamin B2, and vitamin B6. The content of most amino acids in the TWW after *Rhodotorula glutinis* fermentation was greatly reduced, but the content of serine and glycine was somewhat increased. In addition, we found that the content of potassium and magnesium in TWW was relatively high, reaching 369.00 and 63.80 mg/kg, respectively. After *Rhodotorula glutinis* fermentation, the content of these two elements did not change significantly, while the content of zinc and phosphorus was significantly changed, among which the zinc content increased by 62.30% and the phosphorus content decreased by 42.31%.

**Table 4:** The effect of *Rhodotorula glutinis* on the nutrient content of TWW.

Nutrients	Before fermentation	After fermentation	Amino acids (mg/L)	Before fermentation	After fermentation
Protein (g/100 g)	1.90 ± 0.16	1.11 ± 0.09**	Aspartic acid	4.25 ± 0.38	0.48 ± 0.07^***^
Fat (g/100 g)	0.20 ± 0.02	0.20 ± 0.03	Glutamic acid	6.06 ± 0.67	0.35 ± 0.05^***^
Reducing sugar (g/100 g)	<0.25	<0.25	Serine	<0.05	0.14 ± 0.01
Vitamin B1 (mg/L)	0.12 ± 0.01	0.24 ± 0.03**	Glycine	<0.05	0.23 ± 0.03
Vitamin B2 (mg/L)	0.012 ± 0.0017	0.042 ± 0.0055***	Histidine	2.48 ± 0.34	0.25 ± 0.03^***^
Vitamin B6 (mg/L)	0.127 ± 0.018	0.748 ± 0.087***	Arginine	18.60 ± 1.67	0.19 ± 0.02^***^
Zinc (mg/kg)	0.61 ± 0.055	0.99 ± 0.079**	Threonine	0.73 ± 0.06	<0.05
Potassium (mg/kg)	369.00 ± 44.29	347.00 ± 31.23	Alanine	2.99 ± 0.34	<0.05
Nickel (mg/kg)	0.66 ± 0.073	0.59 ± 0.048	Proline	10.90 ± 1.17	<0.05
Copper (mg/kg)	0.20 ± 0.019	0.19 ± 0.02	Tyrosine	4.80 ± 0.53	1.63 ± 0.19^***^
Magnesium(mg/kg)	63.80 ± 8.29	62.20 ± 8.09	Valine	2.11 ± 0.19	<0.05
Manganese (mg/kg)	0.027 ± 0.0037	0.026 ± 0.0025	Methionine	<0.05	<0.05
Calcium (mg/kg)	1.40 ± 0.16	1.46 ± 0.13	Isoleucine	0.90 ± 0.11	0.055 ± 0.006***
Phosphorus (mg/kg)	26.00 ± 2.34	15.00 ± 1.21**	Leucine	1.47 ± 0.13	0.74 ± 0.10**
Barium (mg/kg)	0.47 ± 0.06	0.48 ± 0.04	Cystine	<0.05	<0.05
Iron (mg/kg)	1.40 ± 0.15	1.46 ± 0.12	Phenylalanine	1.45 ± 0.11	<0.05
			Lysine	1.87 ± 0.16	<0.05

* Indicates significant differences (*p* < 0.05).

** Indicates extremely significant differences (*p* < 0.01).

*** Indicates extremely significant differences (*p* < 0.001).

### 3.2. Effect of different components of culture medium on the fermentation of *Rhodotorula glutinis* in TWW

#### 3.2.1. Effect of glucose on fermentation of *Rhodotorula glutinis* in TWW

**Figure 1:**
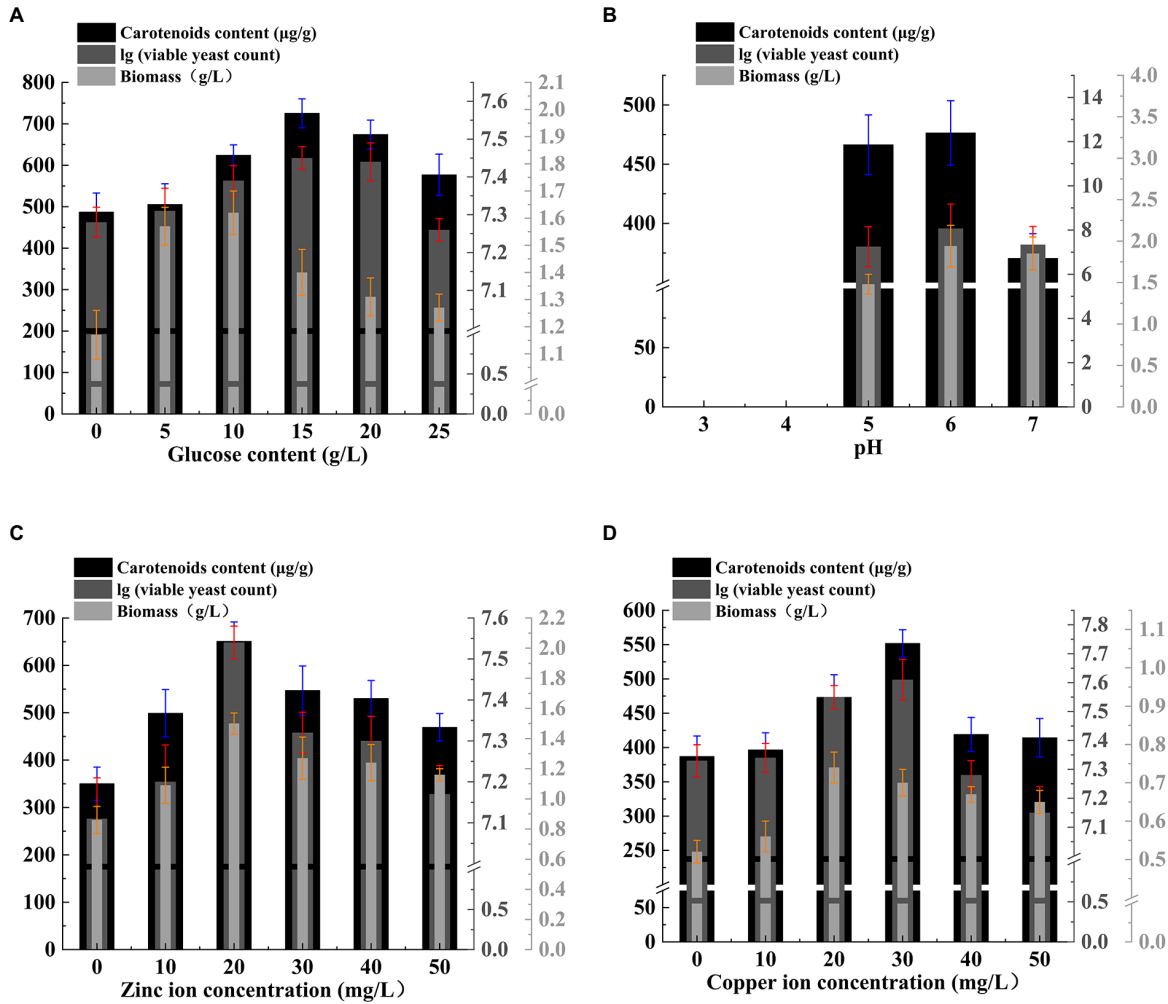
Effect of factors on carotenoids content, biomass and viable yeast count. **(A)** glucose, **(B)** pH, **(C)** zinc ion, **(D)** copper ion.

The growth metabolism of *Rhodotorula glutinis* is largely influenced by the components of the culture matrix. On this basis, we added glucose to the TWW medium to explore the effect of glucose on the growth of *Rhodotorula glutinis*. As can be seen from Figure 1 A, different glucose concentrations had different effects on *Rhodotorula glutinis* biomass, viable yeast count and carotenoids content. When the glucose concentration was 15 g/L, the viable yeast count and carotenoids content of *Rhodotorula glutinis* reached the highest value, which was 2.80 × 10^7^ cfu/ml and 725.36 μg/g, respectively, the carotenoids content was also at a relatively high level.

#### 3.2.2. Effect of pH on fermentation of *Rhodotorula glutinis* in TWW

We set five initial pH as: 3.0, 4.0, 5.0, 6.0, and 7.0 to further investigate the effect of the initial pH on the growth of *Rhodotorula glutinis*. As shown in Figure 1B, the difference in biomass, carotenoids content and viable yeast count of *SRY* were not significant when the initial pH was 4.0 compared to that at an initial pH of 3.0. Then, as the initial pH value increases, these indexes showed a tendency to increase first and then decrease. When the initial pH was 6.0, the *Rhodotorula glutinis SRY* biomass, carotenoids content, and viable yeast count were all high, reaching 1.94 g/L, 476.36 μg/g, and 1.18 × 10^8^ cfu/ml, respectively.

#### 3.2.3. Effect of metal ions on the fermentation of *Rhodotorula glutinis* in TWW

Trace elements can improve the generation of carotenoids in *Rhodotorula glutinis*. We measured the effects of metal ions of different species and concentrations on *Rhodotorula glutinis* biomass, viable yeast count, and carotenoids content. As shown in Figure 1C, the trend of *SRY* biomass, carotenoids content and viable yeast count of *Rhodotorula glutinis* all increased first and then decreased with the increase of zinc ion concentration. When the zinc ion content was 20 mg/L, the maximum values of *SRY* biomass, carotenoids content and viable yeast count were 1.50 g/L, 650.68 μg/g and 3.50 × 10^7^ cfu/ml, respectively. When the manganese ion content was 20 mg/L, the copper ion content was 30 mg/L, and the ferrous iron ion content was 40 mg/L, the *Rhodotorula glutinis SRY* biomass, carotenoids content and viable yeast count were all at higher levels (Figure 1D; Supplementary Figure 1). With the increase of magnesium ion content, both the *Rhodotorula glutinis SRY* biomass and the carotenoids content showed a trend of increase first and then decrease. The maximum *Rhodotorula glutinis SRY* biomass was 1.28 g/L when the magnesium ion content was 20 mg/L, and when the magnesium ion content was 40 mg/L, the maximum *Rhodotorula glutinis SRY* carotenoids content was 621.23 μg/g (Supplementary Figure 1).

### 3.3. Fermentation condition optimization and response surface experiment

By Plackett-Burman test, the *Rhodotorula glutinis* biomass, viable yeast count and carotenoids content in the TWW medium were selected as significantly affected factors (Supplementary Table 3). The test results showed that the initial pH value (A), soybean oil content (B), glucose content (C), and zinc ion content (H) had significant effects on SRY biomass, carotenoids content, and viable yeast count (*p* < 0.05, Supplementary Tables 4–6).

As shown in Tables 5, 6, with overall desirability as the response value, under the selected test conditions selected, the *p* value of factors A, B, C, H, AH, A^2^, B^2^, C^2^, and H^2^ were less than 0.001, indicates that they have a very significant effect on the desirability of *Rhodotorula glutinis* fermentation condition optimization, and the factor AB has a significant effect (0.01 < *p* < 0.05). The value of “Lack of Fit” was 0.6353, inferring that “Lack of Fit” was not significant relative to the pure error (*p* > 0.05); therefore, the model was reliable. The R^2^ was 0.9846, indicating that the total variable of response values over 98.46% could be represented by this model. The Adj.R^2^ was 0.9692, confirming that the model reflected the relationship between each single factor, with a high fitting degree and small experimental error. The fit of the quadratic multivariate equation of *SRY* fermentation is as follows:


$$\begin{array}{c} \mathrm{Overall desirability}=0.47+0.065\times A-0.020\times B+0.016\times C\\ +0.015\times D+0.017\times A\times B+0.010\times A\times C\\ +0.027\times A\times D+0.001825\times B\times C\\ +0.005418\times B\times D-0.012\times C\times D-0.056\times A^{2}\\ -0.045\times B^{2}-0.028\times C^{2}-0.079\times D^{2} \end{array}$$

The secondary multiple regression model was optimized, and the optimal fermentation conditions were as follow: the initial pH was 6.40, soybean oil content was 0.80 ml/L, glucose content was 16.32 g/L, and zinc ion content was 20.52 mg/L, predicting the overall desirability of 0.492 under the above conditions. Three parallel tests were carried out according to the best fermentation conditions obtained in the experiment, and the average value of the fermentation overall desirability was 0.471 ± 0.03, which was close to the theoretical predicted value, indicating that the model can better reflect the actual situation of the fermentation condition optimization of *Rhodotorula glutinis*. The optimized *Rhodotorula glutinis* fermentation condition obtained biomass was 2.23 g/L, the carotenoids content was 832.86 μg/g, and the viable yeast count was 7.08 × 10^7^ cfu/ml.

**Table 5:** Response surface methodology results of process optimization.

No.	A	B	C	H	Biomass (g/L)	Carotenoids content (μg/g)	l g (Viable yeast count)	Overall desirability
1	0	−1	1	0	2.05 ± 0.18	740.73 ± 60.26	7.79 ± 0.05	0.43
2	1	0	0	1	2.09 ± 0.19	796.16 ± 71.62	7.81 ± 0.04	0.44
3	1	1	0	0	2.07 ± 0.15	746.19 ± 59.51	7.78 ± 0.04	0.43
4	0	0	0	0	2.34 ± 0.24	858.92 ± 77.31	7.91 ± 0.06	0.49
5	1	0	0	−1	1.79 ± 0.16	646.30 ± 58.17	7.72 ± 0.05	0.37
6	−1	−1	0	0	1.61 ± 0.17	606.59 ± 66.68	7.68 ± 0.03	0.34
7	0	0	1	−1	1.76 ± 0.17	633.59 ± 68.69	7.73 ± 0.04	0.37
8	0	0	−1	−1	1.50 ± 0.14	520.60 ± 46.85	7.64 ± 0.03	0.31
9	0	1	−1	0	1.73 ± 0.17	633.30 ± 63.22	7.72 ± 0.05	0.37
10	−1	0	−1	0	1.49 ± 0.13	521.04 ± 47.03	7.63 ± 0.04	0.31
11	1	0	−1	0	1.97 ± 0.17	719.53 ± 69.07	7.77 ± 0.03	0.41
12	0	−1	−1	0	1.95 ± 0.18	714.60 ± 78.61	7.75 ± 0.04	0.41
13	−1	0	0	−1	1.36 ± 0.11	447.60 ± 60.66	7.62 ± 0.06	0.28
14	1	0	1	0	2.31 ± 0.22	823.46 ± 97.86	7.85 ± 0.05	0.47
15	0	0	0	0	2.11 ± 0.18	838.59 ± 96.44	7.86 ± 0.04	0.46
16	0	1	0	−1	1.41 ± 0.12	506.48 ± 46.58	7.63 ± 0.03	0.30
17	0	0	1	1	1.85 ± 0.19	685.20 ± 81.54	7.76 ± 0.05	0.39
18	1	−1	0	0	2.08 ± 0.27	748.60 ± 69.37	7.79 ± 0.03	0.43
19	0	0	0	0	2.18 ± 0.28	831.55 ± 99.77	7.84 ± 0.04	0.46
20	0	0	0	0	2.23 ± 0.24	836.59 ± 75.29	7.83 ± 0.04	0.47
21	0	−1	0	−1	1.68 ± 0.21	616.95 ± 54.53	7.69 ± 0.04	0.35
22	−1	0	0	1	1.15 ± 0.11	396.55 ± 37.67	7.53 ± 0.03	0.24
23	−1	0	1	0	1.54 ± 0.17	577.22 ± 63.40	7.68 ± 0.03	0.33
24	0	0	0	0	2.19 ± 0.18	843.57 ± 75.93	7.86 ± 0.04	0.47
25	−1	1	0	0	1.35 ± 0.11	415.48 ± 36.39	7.62 ± 0.05	0.27
26	0	-1	0	1	1.78 ± 0.19	640.60 ± 69.47	7.74 ± 0.05	0.37
27	0	1	0	1	1.57 ± 0.22	603.57 ± 53.72	7.69 ± 0.04	0.34
28	0	1	1	0	1.86 ± 0.17	692.40 ± 76.18	7.74 ± 0.03	0.39
29	0	0	−1	1	1.84 ± 0.20	656.50 ± 60.75	7.73 ± 0.04	0.38

**Table 6:** Variance analysis of the response surface methodology.

Source of variance	Mean square	*F* value	*p* value	Significant
Model	0.00869	64.01	< 0.0001	**
A-pH	0.05000	368.33	< 0.0001	**
B-soybean oil	0.00458	33.74	< 0.0001	**
C-glucose	0.00321	23.65	0.0003	**
H-zinc ion	0.00276	20.35	0.0005	**
AB	0.00114	8.39	0.0117	*
AC	0.00043	3.2	0.0952	
AH	0.00298	21.94	0.0004	**
BC	0.00001	0.098	0.7587	
BH	0.00012	0.86	0.3682	
CD	0.00056	4.16	0.0608	
A^2^	0.02100	152.05	< 0.0001	**
B^2^	0.01300	97.2	< 0.0001	**
C^2^	0.00500	36.85	< 0.0001	**
H^2^	0.04000	297.4	< 0.0001	**
Lack of fit	0.00013	0.82	0.6353	not significant
R^2^	0.9846			
Adj. R^2^	0.9692			

* Indicates significant differences (*p* < 0.05).

** Indicates extremely significant differences (*p* < 0.01).

### 3.4. Influence of each factor on the overall desirability of the response value

**Figure 2:**
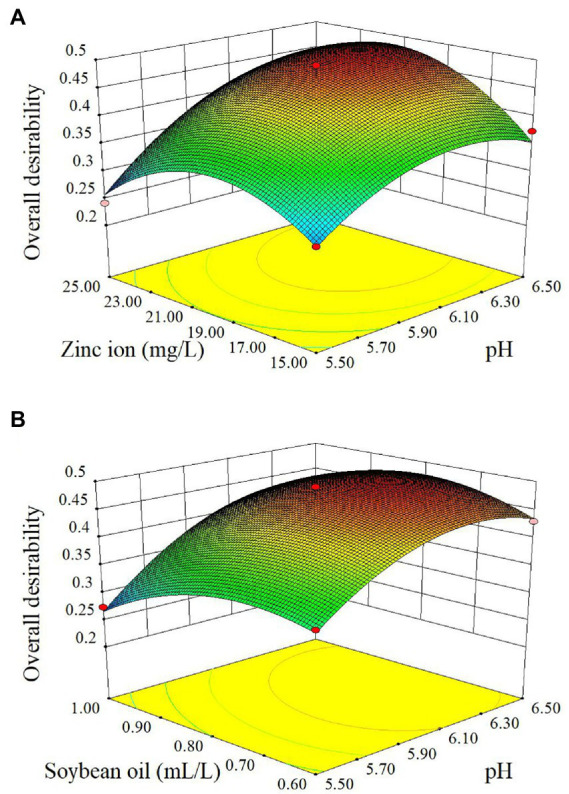
The effect of factors on overall desirability with response values. **(A)** Effect of pH and zinc ion content on overall desirability with *Rhodotorula glutinis* fermentation process; **(B)** Effect of pH and soybean oil content on overall desirability with *Rhodotorula glutinis* fermentation process.

The effects of the initial pH and zinc ion content on the overall desirability of the *Rhodotorula glutinis* fermentation condition, and the effects of the initial pH versus soybean oil content on the overall desirability of the *Rhodotorula glutinis* fermentation condition are shown in Figure 2. The results showed that the initial pH value and zinc ion content and soybean oil content significantly affected the overall desirability of *Rhodotorula glutinis* fermentation condition, which was consistent with the significance test results of the overall desirability partial regression coefficient.

### 3.5. The effect of *Rhodotorula glutinis* using TWW on laying hens

#### 3.5.1. Effect of *Rhodotorula glutinis* on the laying performance of laying hens

**Figure 3:**
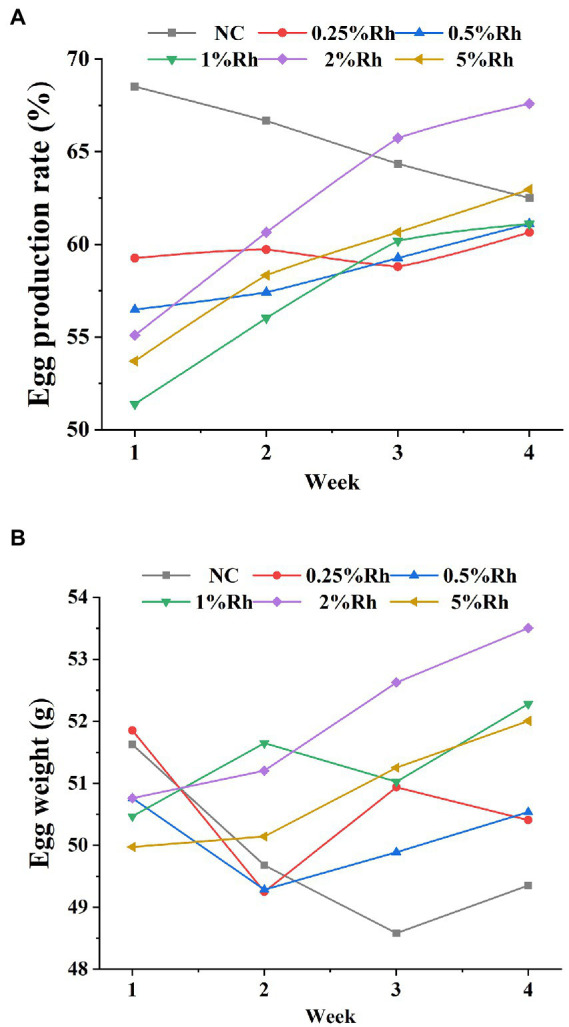
Effect of *Rhodotorula glutinis* fermented in TWW on the productive performance of laying hens. **(A)** egg production rate; **(B)** egg weight.

As shown in the Figure 3, the laying rate and egg weight of laying hens fed normal diet gradually decreased with time, while the laying rate of other groups gradually increased with time, especially for laying hens fed 2% *Rhodotorula glutinis* diet, the laying rate and egg weight of this group increased significantly in the fourth week. The results showed that, the laying rate of laying hens decreased gradually as the weather turned cooler, and feeding the *Rhodotorula glutinis* feed could change this trend to some extent.

#### 3.5.2. Effect of *Rhodotorula glutinis* cultured with TWW on egg quality

**Figure 4:**
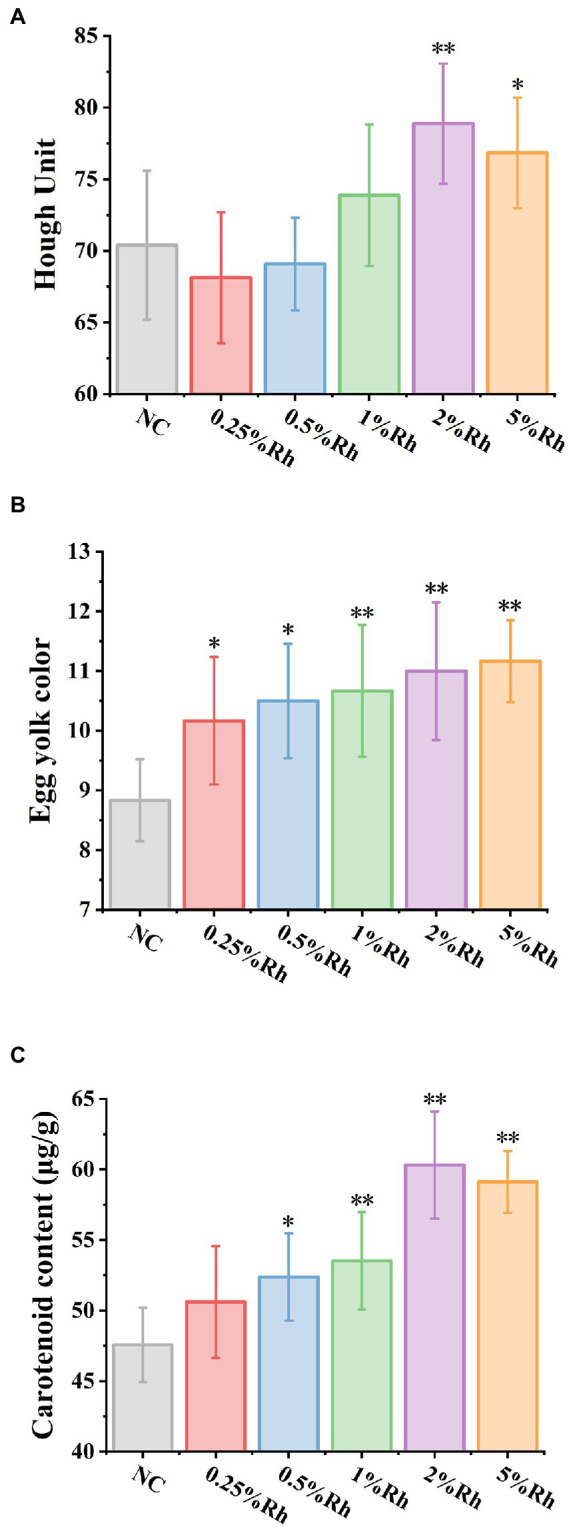
The effect of *Rhodotorula glutinis* fermented in TWW on egg quality. **(A)** Hough unit; **(B)** egg yolk color; **(C)** carotenoids content. *indicates significant differences compared with NC group (*p* < 0.05); **indicates extremely significant differences compared with NC group (*p* < 0.01). Values represent mean ± standard deviation (*n* = 6).

According to the Figure 4, Haugh unit of 2 and 5% *Rhodotorula glutinis* in laying hens had significantly higher than those in the NC group (*p* < 0.05). The Haugh unit was determined by the relationship between the height and the weight of the albumin. The Haugh unit is a parameter indicative of the egg quality. Our results indicated that the yolk color and carotenoids content in all *Rhodotorula glutinis* treated groups were significantly higher than those in untreated groups (*p* < 0.05).

## 4. Discussion

When analyzing the composition of the TWW, we found abundant potassium and magnesium elements, which could promote the accumulation of *Rhodotorula glutinis* biomass and carotenoids. Study by Aleksandr et al. showed that the addition of K^+^ and Na^+^ to *Rhodotorula toruloides* cultures induced glucose utilization and increased β-carotene production by 60% (32). El-Banna et al. showed that the highest biomass of *Rhodotorula glutinis* var. *glutinis* was obtained in a medium containing Mg^2+^ (33). After the fermentation of TWW by *Rhodotorula glutinis*, the zinc content increases greatly, and the content of phosphorus decreases. Sara et al. showed that the amount of zinc in the yeast-fermented bread was greatly increased (17.49–22.89%) (34). Zinc can be used as a component of various enzymes to participate in the process of body growth and protein metabolism. Yeast can absorb and transform zinc elements during growth, and make it organically combined with proteins and polysaccharides in yeast, so that zinc can be absorbed and utilized more efficiently and safely. Yeast can absorb phosphorus from fermentation substrates, and Wang et al. showed that after fermented tofu whey, *Schizosaccharomyces Schizochytrium* sp. *S31.* of removal rate of total phosphorus was as high as 59.3% (35). Our study also found that *Rhodotorula glutinis* produced more vitamins B1, B2 and B6 after fermentation with TWW, which may be due to the production of multiple vitamins during fermentation of *Rhodotorula glutinis*. B vitamins are reported to improve the biosynthesis of yeast and carotenoids of *Rhodotorula gracilis* and *Rhodotorula mucilaginosa*, which creates a virtuous cycle of *Rhodotorula glutinis* reproduction in TWW (36, 37). The content of most amino acids in the TWW decreased significantly after fermentation, which may be due to the ability of amino acids to enter the *Rhodotorula glutinis* cells through the transmembrane transport mechanism and become the nitrogen source for its growth and reproduction.

According to the composition changes of TWW before and after the action of *Rhodotorula glutinis*, we further explored the effect of metal ion addition in TWW on *Rhodotorula glutinis*. When the zinc ion content increased from 0 to 50 mg/kg, the *Rhodotorula glutinis* biomass, viable yeast count and carotenoids content showed a trend of increasing in the first step and then decreasing. Zinc ions could promote carotenoids production in *Rhodotorula glutinis*, and this promotion might result from the activation of specific desaturases involved in carotenoids synthesis by zinc ions (38–40). As the content of magnesium ions in the TWW medium increases, the *Rhodotorula glutinis* biomass and carotenoids content showed a trend of increase first and then decrease, which may be due to the promoting effect of the appropriate amount of magnesium ions. The study by Mariana et al. showed that magnesium could increase biomass and carotenoids content under substrates like maltose, sucrose and sugarcane juice (41). According to our results, the TWW medium originally contained a lot of magnesium, so we believed that the TWW medium was more suitable as a substrate for carotenoids production by *Rhodotorula glutinis*. In our study, Mn^2+^, Cu^2+^, and Fe^2+^ had effectively promoted the generation of carotenoids, which was consistent with previous studies (42, 43). Based on our results of the nutrient composition of TWW, we found the extremely low amount of reducing sugars in TWW. In contrast to sucrose, lactose, maltose, and fructose, glucose was the most suitable carbon source for producing carotenoids for *Rhodotorula* sp. *RY1801*, as found by Zhao et al. (44). Therefore, we selected glucose as the carbon source for the growth of *Rhodotorula glutinis* in the TWW medium, and found that the best fermentation effect was achieved when the glucose concentration was 15 g/L. Moreover, our study showed that when the initial pH value of the TWW medium was between 5.0 and 6.0, the *Rhodotorula glutinis SRY* biomass, carotenoids content and viable yeast count were all at high levels. *Rhodotorula glutinis* is suitable for producing carotenoids in weakly acidic environments. And a study by Latha et al. showed that the optimal pH for *Rhodotorula glutinis* DFR-PDY to produce carotenoids is 5.5 (45). Mohammad et al. found that the carotenoids production of *Rhodotorula acheniorum* reached its highest value, 263 mg/L, when pH value of whey ultrafiltrate was 5.5 and the content of ammonium sulfate was 3.5 g/L (46). These results are all consistent with the conclusions obtained in this study.

Based on the univariate experiment of the basic components of TWW medium on *Rhodotorula glutinis SRY* biomass, carotenoids content and viable yeast count, the interaction of four factors with significant influence was explored. The response surface model can directly and accurately describe the interaction between two variables (factors), expressing the influence of different variables (factors) on a specific index (47). The regression equation and variance analysis derived from the response surface showed that the correlation order of each factor on the yield was: medium initial pH value > soybean oil content > zinc ion content > glucose content. Overall, the initial pH value had the greatest effect on the growth of *Rhodotorula glutinis* in tofu whey wastewater, followed by the soybean oil content and the least effect on glucose content.

The *in vivo* effects were verified by feeding laying hens with different concentrations of *Rhodotorula glutinis* bodies grown in TWW medium. Our study showed that the laying performance and egg quality of laying hens improved after feeding different concentrations of *Rhodotorula glutinis*. As the weather turned cool, the egg yield of laying hens gradually decreased, and feeding the *Rhodotorula glutinis* diet could change this trend to some extent. This may be due to the antigenic activity of the *Rhodotorula glutinis* cell wall that would activate the immune barrier of laying hens, prevent physiological stress, inhibit the pathogens by producing antimicrobial compounds and/or competing nutrients or adhesion sites, and stimulate immunity, thereby enhancing body function (48–52). At the same time, the carotenoids produced by *Rhodotorula glutinis* could play an antioxidant role, thus alleviating the intestinal oxidation and inflammation damage caused by laying hens (53). The Haugh unit is one of the indicators to evaluate the egg quality. It is associated with the protein to comprehensively evaluate the egg quality. If it shows a high Haugh unit, it shows the internal quality and better freshness of the egg (54). Our study showed that feeding *Rhodotorula glutinis* significantly enhanced the Haugh unit, probably due to the antioxidant capacity of *Rhodotorula glutinis*. The study by Henrieta et al. indicated that probiotic supplements had a significant effect on Haugh units (55, 56). Williams pointed out that the Haugh unit was a function of the protein index and that the two were highly correlated (57). Numerous studies have shown that supplementing red yeast to the diet of laying hens can improve egg yolk color (18, 58, 59). The improvement of yolk color should be attributed to the increase in carotenoids content, which are currently often used as pigments in layer breeding to regulate yolk color, and *Rhodotorula glutinis* is an important source of carotenoids (17, 60).

## 5. Conclusion

In this study, the nutrient changes of TWW before and after the action of *Rhodotorula glutinis* were analyzed, and based on this, the fermentation conditions of *Rhodotorula glutinis* were optimized through the composition of TWW medium. The content of zinc increased greatly, the content of phosphorus decreased, and the content of vitamin B1, B2, and B6 increased to different degrees in the TWW fermented by *Rhodotorula glutinis*. The optimal fermentation conditions of *Rhodotorula glutinis* in TWW medium were as follow: initial pH was 6.40, glucose content was 16.32 g/L, and zinc ion addition was 20.52 mg/L. Under this condition, the overall desirability was 0.471 ± 0.03, the biomass of *Rhodotorula glutinis* was 2.23 g/L, carotenoids content was 832.86 μg/g, and viable yeast count was 7.08 × 10^7^ cfu/ml. This study confirmed that *Rhodotorula glutinis* using TWW as the fermentation substrate could increase the egg yield of hens and improve egg quality, increased the yolk color and carotenoids content. This not only alleviates the environmental pollution problem caused by the discharge of TWW, but also reduces the cultivation cost of *Rhodotorula glutinis*, and improves the nutritional value of biological feed of laying hens, opening up a new direction for the efficient utilization of TWW.

## Data availability statement

The original contributions presented in the study are included in the article/Supplementary material, further inquiries can be directed to the corresponding authors.

## Ethics statement

The animal study was reviewed and approved by Animal Experiment Ethics Committee, Jilin Academy of Agricultural Sciences.

## Author contributions

JW, MS, DL, and HX contributed to conception and design of the study. XX, WL, and YS performed the statistical analysis. XX wrote the first draft of the manuscript. MH, XM, YC, and HN wrote sections of the manuscript. All authors contributed to the article and approved the submitted version.

## Funding

This research was funded by Innovation Project of Jilin Academy of Agricultural Sciences - Directed Commission (CXGC2021TD102), Changchun Science and Technology Bureau Key Research and Development Project (21ZGN36), and Jilin Province Science and Technology Development Plan (202202051NC).

## Conflict of interest

The authors declare that the research was conducted in the absence of any commercial or financial relationships that could be construed as a potential conflict of interest.

## Publisher’s note

All claims expressed in this article are solely those of the authors and do not necessarily represent those of their affiliated organizations, or those of the publisher, the editors and the reviewers. Any product that may be evaluated in this article, or claim that may be made by its manufacturer, is not guaranteed or endorsed by the publisher.
