# Supplementary material for: Study on the fermentation effect of Rhodotorula glutinis utilizing tofu whey wastewater and the influence of Rhodotorula glutinis on laying hens
Source: Front Nutr. 2023 Feb 24;10:1125720. doi: 10.3389/fnut.2023.1125720 (PMC9998534; doi:10.3389/fnut.2023.1125720)
Supplement: Supplementary file 1 [file Data_Sheet_1.docx]

Supplementary Material

Study on the fermentation effect of Rhodotorula glutinis completely utilizing tofu whey wastewater

Xifei Xu^1,2^, Wenjian Liu^2,3^, Honghong Niu^2^, Mei Hua^2^, Ying Su^2^, Xinyu Miao^2^, Yanping Chi^2^, Hongyan Xu^1*^, Jinghui Wang^2*^, Mubai Sun^2*^, Da Li^2*^

*** Correspondence:**Hongyan Xu, Jinghui Wang, Mubai Sun, Da Li
xuhongyan@ybu.edu.cn (H. Xu), [wjhjaas@cjaas.com](mailto:wjhjaas@cjaas.com) (J. Wang), [623379903@qq.com](mailto:623379903@qq.com) (M. Sun), [Lida@cjaas.com](mailto:Lida@cjaas.com) (D. Li)

# Supplementary Data

Determination of protein content

The protein content was determined by Kjeldahl method. Briefly, the sample (10 g), copper sulfate (0.4 g), potassium sulfate (6 g) and sulfuric acid (20 mL) were added to the digestion tube and mixed well. The digestion tube was heated in the digestion furnace, and continued to be heated for 1 h after the digestion furnace temperature reached 420 °C. The mixture then appeared in a clear blue-green color. The mixture was cooled and removed, mixed with water (50 mL) and the nitrogen content was determined using an automatic Kjeldahl nitrogen tester. Before using the automatic Kjeldahl nitrogen tester, sodium hydroxide solution (400 g/L), hydrochloric acid standard titration solution (0.05 mol/L) and boric acid solution containing mixed indicator (methyl red ethanol solution: methylene blue ethanol solution = 2:1, ready to use) were added. The protein content was calculated as follows:

Protein content (g/100g) = ((V_1_-V_2_)×c×0.014/(m×V_3_/100))×F×100 (1)

where V_1_ represents the volume of hydrochloric acid standard titration solution consumed by the sample, mL; V_2_ represents the volume of hydrochloric acid standard titration solution consumed by the blank group, mL; c represents the concentration of hydrochloric acid standard titration solution; m represents the mass of the sample, g; V_3_ represents the volume of the digested liquid used, mL; F represents the coefficient of conversion of nitrogen to protein.

Determination of fat content

The fat content was determined by Soxhlet extraction method. The sample (5 g) was added to quartz sand (20 g), evaporated with a boiling water bath and then was dried (100 °C, 30 min). The mixture was ground and transferred to a filter paper cartridge in a Soxhlet extractor. The dry receiver bottle was attached to the Soxhlet extractor. Anhydrous ether was added to two-thirds of the receiving flask from the upper end of the extractor. The anhydrous ether was extracted continuously at reflux by heating in a water bath (6 times/h, 10 h). At the end of the extraction, anhydrous ether was recovered until the volume of solvent remaining in the receiving vial was 1 mL. The remaining solvent was dried (100 °C, 1 h) after being evaporated in a water bath, and then was weighed (after cooling). The fat content was calculated as follows:

Fat content (g/100 g) = (m_1_-m_0_)/m_2_×100 (2)

where m_1_ represents the content of the receiver bottle and fat after drying, g; m_0_ represents the mass of the receiver bottle, g; m_2_ represents the mass of the sample, g.

Determination of sugar content

The reducing sugar content was determined by the potassium permanganate titration method. The sample (25 g), water (50 mL), alkaline copper tartrate A solution (34.639 g of copper sulfate and 0.5 mL of sulfuric acid were fixed with water to 500 mL, 10 mL) and sodium hydroxide solution (40 g/L, 4 mL) were mixed and fixed to 250 mL. The mixture was allowed to stand for 30 min, after which it was filtered. The filtrate (50 mL), basic copper tartrate A solution (25 mL) and basic copper tartrate B solution (173 g of potassium sodium tartrate and 50 g of sodium hydroxide were fixed with water to 500 mL, 25 mL) were added to a 500 mL beaker and mixed. The beaker was covered by a watch glass, heated to boiling (within 4 min) and then boiled (2 min) and filtered by extraction with a Gooch crucible. The beaker and the precipitate were washed with hot water at 60 °C until the washings were not alkaline. The Gooch crucible, iron sulfate solution (50 g/L, 25 mL) and water (25 mL) were put back into the 500 mL beaker mentioned above, stirred until the cuprous oxide was completely dissolved and titrated with potassium permanganate standard solution (0.1 mol/L) until slightly red as the end point. Water (50 mL) was determined by the same method and considered as a blank group. The reducing sugars content was calculated as follows:

Reducing sugar content (g/100 g) = (V-V_0_)×c×71.54×25/(m×V_1_) (3)

where V represents the volume of potassium permanganate standard solution consumed by the sample, mL; V_0_ represents the volume of potassium permanganate standard solution consumed by the blank group, mL; c represents the concentration of potassium permanganate standard solution, mol/L; m represents the mass of the sample, g; V_1_ represents the volume of the sample, mL.

Determination of mineral elements content

Mineral elements content was determined by inductively coupled plasma-mass spectrometry (ICP-MS). Briefly, the sample (3.00 mL) with nitric acid (10 mL) was added to the microwave digestion inner tank and left overnight with the lid on. After the digestion, the inner lid was rinsed with a small amount of water and the digestion tank was placed in an ultrasonic water bath for sonication (5 min), after which it was fixed to 50 mL with water. Blank experiments were performed in the same way. The signal response values of the elements and the internal standard elements of the mixed standard solutions to be measured were determined by an inductively coupled plasma mass spectrometer and the standard curves were plotted. The mineral element content was calculated as follows:

Element content (mg/kg) = (ρ-ρ_0_)×V×f/m (4)

where ρ represents the mass concentration of the measured element in the sample, mg/L; ρ_0_ represents the mass concentration of the measured element in the blank experiment, mg/L; V the fixed volume of the sample digest, mL; f represents the dilution multiple of the sample; m represents the volume of the sample, mL.

Determination of mineral vitamin B1 content

The sample (20 g) and hydrochloric acid solution (0.1 mol/L, 60 mL) were well mixed in a 100 mL conical flask, capped with a soft stopper and autoclaved (121 °C, 30 min). After sterilization, the mixture was cooled to 40 °C. The pH of the mixture was adjusted to 4.0 by sodium acetate solution (2.0 mol/L). The mixed enzyme solution (2.0 mL) and the mixture were mixed and left overnight (16 h) in an incubator (37 °C). The enzymatic solution was fixed with water to 100 mL, filtered, and the filtrate was the extract solution. The extract solution (2.0 mL) and the basic potassium ferricyanide solution (1.0 mL) were vortexed and mixed, then n-butanol (2.0 mL) was added, vortexed and mixed again (1.5 min) and left to stand (10 min). After partitioning of the mixture, the upper liquid layer was filtered through an organic microporous membrane (0.45 μm). The filtrate was placed in a brown inlet vial (2 mL) and set aside. The standard series solution (2.0 mL) was treated in the same way. Chromatographic column: C_18_ reversed-phase column (particle size 5 μm, 250 mm×4.6 mm); mobile phase: sodium acetate solution (0.05 mol/L)-methanol (65:35); flow rate: 0.8 mL/min; injection volume: 20 μL. Detection wavelength: excitation wavelength 375 nm, emission wavelength 435 nm. The peak area of vitamin B1 was determined by high performance liquid chromatography. The standard curve was plotted with the concentration of the standard solution (μg/mL) as the horizontal coordinate and the peak area as the vertical coordinate. The peak areas of the samples for vitamin B1 were determined by high performance liquid chromatography. The concentration of vitamin B1 in the sample was calculated from the standard curve. The vitamin B1 content was calculated as follows:

Vitamin B1 content (mg/100g) = c×V×f/(m×10) (5)

where c represents the concentration of vitamin B1 in the sample calculated from the standard curve, μg/mL; V represents the volume of the fixed volume of the extract, mL; f represents the volume of the fixed volume of the filtrate, mL; m represents the mass of the sample, g.

Determination of mineral vitamin B2 content

The sample (10 g) and hydrochloric acid solution (0.1 mol/L, 60 mL) were well mixed in a 100 mL conical flask, capped with a soft stopper and autoclaved (121 °C, 30 min). After sterilization, the mixture was cooled to 40 °C. The pH of the mixture was adjusted to 6.0 by sodium hydroxide solution (1.0 mol/L). The mixture and the mixed enzyme solution (2 mL) were mixed and digested overnight (37 °C). The enzyme solution was fixed with water to 100 mL and then filtered through an aqueous phase filter membrane (0.45 μm). Blank samples were manipulated in the same way. Chromatographic column: C_18_ reversed-phase column (particle size 5 μm, 150 mm×4.6 mm); mobile phase: sodium acetate solution (0.05 mol/L)-methanol (65:35); flow rate: 1 mL/min; column temperature: 30 ℃; injection volume: 20 μL. Detection wavelength: excitation wavelength 462 nm, emission wavelength 522 nm. The peak areas of vitamin B2 were determined by high performance liquid chromatography. The standard curve was plotted with the concentration of the standard solution (μg/mL) as the horizontal coordinate and the peak area as the vertical coordinate. The peak areas of vitamin B2 of the samples were determined by high performance liquid chromatography. The vitamin B2 concentration in the samples was calculated from the standard curve. The vitamin B2 content was calculated as follows:

Vitamin B2 content (mg/100 g) = ρ×V×0.1/m (6)

where ρ represents the concentration of vitamin B2 in the sample calculated from the standard curve, μg/mL; V represents the final volume of the sample fixation, mL; m represents the sample mass, g.

Determination of mineral vitamin B6 content

The samples (20 g) were allowed to stand for 10 min in a 150 mL conical flask. The pH of the samples was adjusted to 1.7 and then left for 1 min. Afterwards, the pH of the samples was adjusted to 4.5 by sodium hydroxide solution. The above-mentioned conical flasks were shaken with ultrasound (10 min). The samples were fixed with water to 50 mL and then filtered through a microporous membrane (0.45 μm) and the filtrate was collected. Chromatographic column: C18 reversed-phase column (particle size 5 μm, 150 mm×4.6 mm). Mobile phase: methanol (50 mL), sodium octanesulfonate (2.0 g) and triethylamine (2.5 mL) were fixed with water to 1000 mL, after which the pH was adjusted to 3.0 by glacial acetic acid. The mixture was filtered through a microporous membrane (0.45 μm) and the filtrate was collected. Flow rate: 1 mL/min; column temperature: 30 ℃; injection volume: 20 μL. Detection wavelength: excitation wavelength 293 nm, emission wavelength 395 nm. The peak area of vitamin B6 was determined by high performance liquid chromatography. The standard curve was plotted with the concentration of the standard solution (μg/mL) as the horizontal coordinate and the peak area as the vertical coordinate. The peak areas of vitamin B6 of the samples were determined by high performance liquid chromatography. The concentration of vitamin B6 in the sample was calculated from the standard curve. The vitamin B6 content was calculated as follows:

Each component content in vitamin B6 (mg/100 g) = ρ×V×0.1/m (7)

Vitamin B6 content (mg/100 g) = X_1_+X_2_×1.012+X_3_×1.006 (8)

where ρ represents the concentration of each component of vitamin B6 in the sample calculated from the standard curve, μg/mL; V represents the final volume of the sample for fixing, mL; m represents the mass of the sample, g; X_1_ represents the content of pyridoxine in the sample, mg/100 g; X_2_ represents the content of pyridoxal in the sample, mg/100 g; X_3_ represents the content of pyridoxamine in the sample, mg/100 g.

Determination of free amino acid content.

Chromatographic column: UG120 C_18_ (4.6 mm×250 mm×5 μm), detection wavelength: 254 nm, column temperature: 40 ℃, injection volume: 10 μL, mobile phase A: 0.1 mol/L anhydrous sodium acetate: acetonitrile = 97: 3 (V/V), mix well and adjust the pH to 6.5 with glacial acetic acid; mobile phase B: acetonitrile: water = 80: 20 (V/V). The standard solution (100 μg/mL, 0.1 mL) of 17 amino acid mixture was measured in a 5 mL centrifuge tube and dried in a vacuum drying oven at 60 ℃. The derivatization reagent was prepared (ethanol:phenyl isothiocyanate: water: triethylamine= 7: 1: 1: 1, V/V/V/V/V), ready to use, and filled with nitrogen during preparation. Nitrogen was charged into the sample centrifuge tube, derivatization reagent (50 μL) was added accurately, and derivatization was carried out at room temperature for 30 min. Mobile phase A (0.45 mL) was added to the sample centrifuge tube, mixed and then filtered for measurement. Standards of 17 amino acids (20 μg/mL) were measured after derivatization.

# Supplementary Figures and Tables

## Supplementary Figures


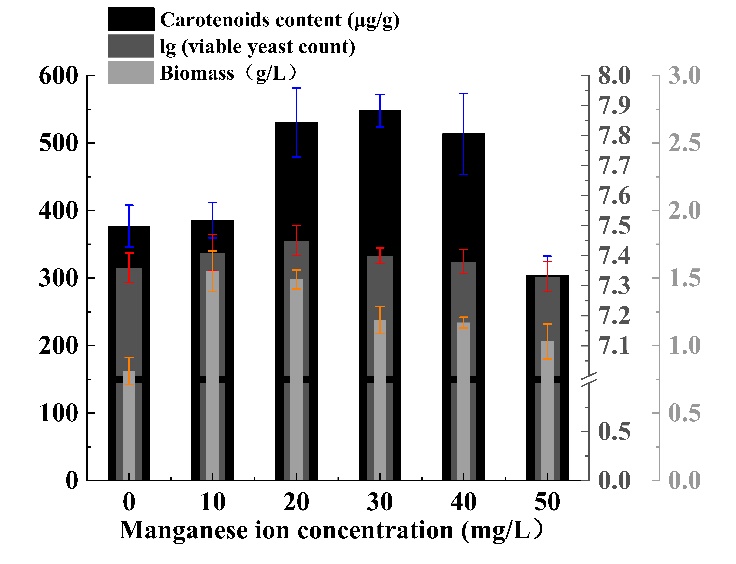

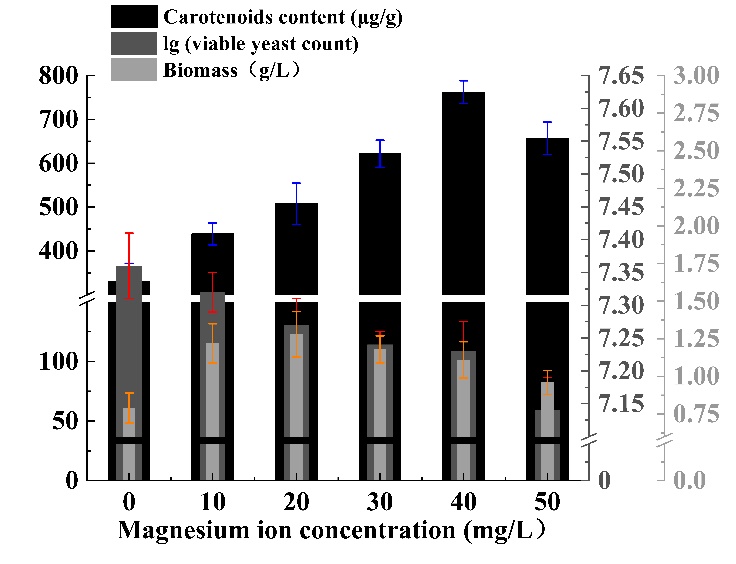


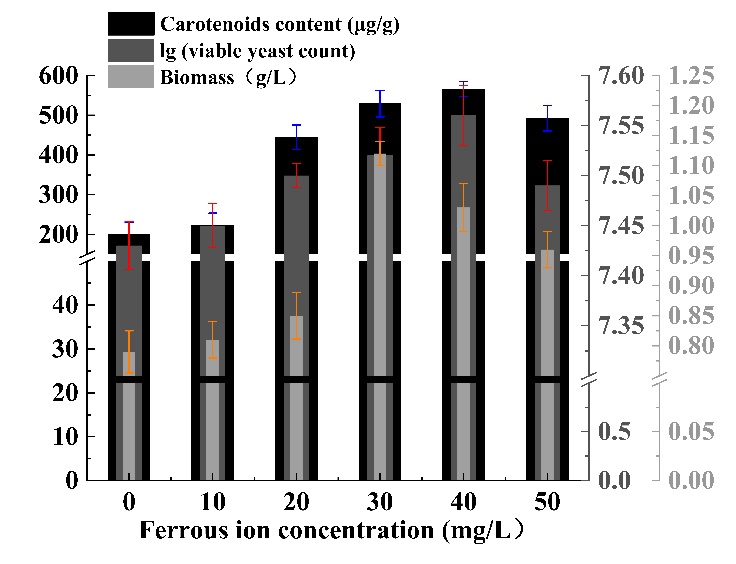


**Supplementary Figure 1.** Effect of factors on carotenoids content, biomass and viable yeast count (A) manganese ion, (B) magnesium ion, (C) ferrous ion.

## Supplementary Tables

**Supplementary Table 1.** Design of single factor experiment.

| Factor | -2 | -1 | 0 | +1 | +2 |
| --- | --- | --- | --- | --- | --- |
| PH | 3 | 4 | 5 | 6 | 7 |
| Glucose (g/L) | 5 | 10 | 15 | 20 | 25 |
| Manganese ion (mg/L) | 10 | 20 | 30 | 40 | 50 |
| Cupric ion (mg/L) | 10 | 20 | 30 | 40 | 50 |
| Magnesium ion (mg/L) | 10 | 20 | 30 | 40 | 50 |
| Zinc ion (mg/L) | 10 | 20 | 30 | 40 | 50 |
| Ferrous ion (mg/L) | 10 | 20 | 30 | 40 | 50 |

**Supplementary Table 2.** Design of Plackett-Burman experiment.

| Factor | -1 | +1 |
| --- | --- | --- |
| A-pH | 5 | 7 |
| B-soybean oil (ml/L) | 0.4 | 1.2 |
| C-glucose (g/L) | 10 | 20 |
| D-hydrogen peroxide (mg/L) | 1 | 1.8 |
| E-vitaminB_1_ (mg/L) | 1.6 | 2 |
| F-manganese ion (mg/L) | 10 | 30 |
| G-magnesium ion (mg/L) | 20 | 40 |
| H-zinc ion (mg/L) | 10 | 30 |

**Supplementary Table 3.** Plackett-Burman test results of fermentation process optimisation.

| No. | A | B | C | D | E | F | G | H | Biomass (g/L) | Carotenoids content (μg/g) | lg (Viable  yeast count) |
| --- | --- | --- | --- | --- | --- | --- | --- | --- | --- | --- | --- |
| 1 | 1 | 1 | 1 | -1 | -1 | -1 | 1 | -1 | 0.53±0.05 | 323.465±29.112 | 7.23±0.05 |
| 2 | -1 | -1 | 1 | -1 | 1 | 1 | -1 | 1 | 0.88±0.07 | 464.454±32.269 | 7.45±0.04 |
| 3 | 1 | -1 | 1 | 1 | 1 | -1 | -1 | -1 | 0.77±0.04 | 375.547±27.168 | 7.39±0.04 |
| 4 | -1 | -1 | 1 | -1 | 1 | 1 | 1 | -1 | 0.48±0.03 | 317.963±29.457 | 7.17±0.06 |
| 5 | 1 | 1 | 1 | 1 | -1 | 1 | 1 | 1 | 1.01±0.08 | 537.654±46.256 | 7.52±0.03 |
| 6 | -1 | -1 | -1 | 1 | -1 | 1 | 1 | -1 | 0.83±0.05 | 395.945±40.058 | 7.41±0.05 |
| 7 | 1 | 1 | -1 | 1 | 1 | 1 | -1 | -1 | 0.73±0.06 | 342.374±33.694 | 7.37±0.04 |
| 8 | 1 | -1 | -1 | -1 | 1 | -1 | 1 | 1 | 1.36±0.11 | 705.243±71.842 | 7.63±0.05 |
| 9 | 1 | 1 | -1 | -1 | -1 | 1 | -1 | 1 | 1.32±0.10 | 620.913±60.447 | 7.61±0.06 |
| 10 | -1 | 1 | 1 | 1 | -1 | -1 | -1 | 1 | 0.81±0.09 | 386.779±39.743 | 7.42±0.05 |
| 11 | -1 | -1 | -1 | -1 | -1 | -1 | -1 | -1 | 0.93±0.03 | 502.37±51.901 | 7.46±0.04 |
| 12 | -1 | 1 | -1 | 1 | 1 | -1 | 1 | 1 | 0.86±0.06 | 402.579±32.119 | 7.45±0.03 |

**Supplementary Table 4.** Analysis of variance with biomass as response value.

| Source of variance | Mean square | F value | P value | Significant |
| --- | --- | --- | --- | --- |
| A-pH | 0.10 | 43.59 | 0.0051 | ** |
| B-soybean oil | 0.15 | 69.28 | 0.0036 | ** |
| C-glucose | 0.12 | 56.88 | 0.0048 | ** |
| D-hydrogen peroxide | 0.20 | 91.68 | 0.0024 | ** |
| E-vitaminB_1_ | 0.00 | 0.10 | 0.7729 |  |
| F-manganese ion | 0.06 | 25.53 | 0.015 | * |
| G-magnesium ion | 0.00 | 0.00 | 0.9546 |  |
| H-zinc ion | 0.01 | 5.22 | 0.1064 |  |
| Residual | 0.44 | 201.29 | 0.0008 | *** |
| R^2^ | 0.9915 | | | |
| Adj. R^2^ | 0.9687 | | | |

*indicates significant differences (*p* < 0.05); **indicates extremely significant differences (*p* < 0.01).

**Supplementary Table 5.** Analysis of variance with carotenoids content as response value.

| Source of variance | Mean square | F value | P value | Significant |
| --- | --- | --- | --- | --- |
| A-pH | 19893.51 | 10.78 | 0.038 | * |
| B-soybean oil | 35587.35 | 19.29 | 0.0219 | * |
| C-glucose | 32843.14 | 17.80 | 0.0243 | * |
| D-hydrogen peroxide | 26466.83 | 14.34 | 0.0323 | * |
| E-vitaminB_1_ | 3143.97 | 1.70 | 0.2829 |  |
| F-manganese ion | 13427.42 | 7.28 | 0.0739 |  |
| G-magnesium ion | 23.18 | 0.01 | 0.9178 |  |
| H-zinc ion | 7.66 | 0.00 | 0.9527 |  |
| Residual | 90362.93 | 48.98 | 0.006 | ** |
| R^2^ | 0.9664 | | | |
| Adj. R^2^ | 0.8768 | | | |

*indicates significant differences (*p* < 0.05); **indicates extremely significant differences (*p* < 0.01).

**Supplementary Table 6.** Analysis of variance with viable yeast count as response value.

| Source of variance | Mean square | F value | P value | Significant |
| --- | --- | --- | --- | --- |
| A-pH | 0.024 | 30.77 | 0.0085 | ** |
| B-soybean oil | 0.031 | 39.06 | 0.0083 | ** |
| C-glucose | 0.031 | 39.91 | 0.008 | ** |
| D-hydrogen peroxide | 0.047 | 59.8 | 0.0045 | ** |
| E-vitaminB_1_ | 0.01 | 7.15 | 0.0755 |  |
| F-manganese ion | 0.015 | 19.07 | 0.0222 | * |
| G-magnesium ion | 0.00 | 0.27 | 0.6418 |  |
| H-zinc ion | 0.01 | 8.94 | 0.0581 |  |
| Residual | 0.12 | 155.3 | 0.0011 | ** |
| R^2^ | 0.9880 | | | |
| Adj. R^2^ | 0.9558 | | | |

*indicates significant differences (p < 0.05); **indicates extremely significant differences (*p* < 0.01).
